# A taxonomic study on the genus *Ettchellsia* Cameron, with descriptions of three new species (Hymenoptera, Megalyridae, Dinapsini)

**DOI:** 10.3897/zookeys.254.4182

**Published:** 2012-12-21

**Authors:** Toshiharu Mita, Scott R. Shaw

**Affiliations:** 1 Laboratory of Entomology, Faculty of Agriculture, Tokyo University of Agriculture, 1737 Funako, Atsugi-shi, Kanagawa 243-0034 JAPAN; 2 University of Wyoming Insect Museum, Department of Ecosystem Science and Management (3354), University of Wyoming, 1000 East University Avenue, Laramie, Wyoming 82071, U.S.A.

**Keywords:** Taxonomy, parasitic wasps, South East Asia

## Abstract

Three new species of *Ettchellsia* Cameron, namely, *Ettchellsia ignita* **sp. n.** from Peninsular Malaysia and Borneo, *Ettchellsia nigripes* **sp. n.** from Sulawesi and *Ettchellsia reidi* **sp. n.** from Borneo are described and illustrated. A key to the species of *Ettchellsia* is provided based on females.

## Introduction

Cameron (1909) described a remarkable new wasp genus from Borneo that he dedicated to his housekeeper, Mary Ettchells. Cameron found the insect so unusual that he struggled to place it to family, and debated if it should be placed into a new family. Subsequent authors (Baltazar 1961; Shaw 1988, 1990; Vilhelmsen et al. 2010) have clarified the placement of *Ettchellsia* Cameron in the family Megalyridae. *Ettchellsia* Cameron 1909 is a small genus comprising three described species (Cameron 1909; Baltazar 1961; He 1991). *Ettchellsia* species show an Indomalayan distribution, i.e., *Ettchellsia piliceps* Cameron occurring in Borneo, *Ettchellsia philippinensis* Baltazar occurring in Philippines and *Ettchellsia sinica* occurring in Yunnan, China. Shaw (1990) defined *Ettchellsia* as a monophyletic genus based on three synapomorphies: the rugose hind tibia, unique pattern of propodeal carinae (as in Figs 10–12), and smooth posterior border of the mesopleuron, without a row of foveae separating the mesopleuron and metapleuron. Shaw (1990) also examined specimens from Thailand and Viet Nam, which he regarded as range extensions and variations of *Ettchellsia piliceps* Cameron. One specimen from Taiwan was not placed to species because it was a male. Vilhelmsen et al. (2010) also referred to unidentified species of *Ettchellsia* from Taiwan and Thailand. According to recent phylogenetic studies, the genus *Dinapsis* Waterson (distributed in the Afrotropical region) was strongly suggested to be the closest relative of *Ettchellsia* (Shaw 1990; Vilhelmsen et al. 2010). *Ettchellsia* species are quite rare; however, recently some specimens were obtained from Peninsular Malaysia, Sulawesi and Kalimantan. They were identified by us as three different new species. These findings and recent taxonomic works on the genus *Carminator* Shaw, the other genus of the family found in Asia (Mita et al. 2007; Mita and Konishi 2011) may imply the megalyrid species diversity of the Indomayan region is still not clarified well. The biology of *Ettchellsia* has remained unknown, and no distributional areas are reported except for type localities. It is almost the same case with *Carminator*. Based on a knowledge of other Megalyridae, *Ettchellsia* is presumed to be an idiobiont ectoparasitoid, probably attacking beetle larvae (Shaw 1990). Southeast Asia is a key area to understanding the complicated evolutionary history of the family Megalyridae because the sister-group pairs of both *Ettchellsia* (*Dinapsis* Waterson: Afrotropical) and *Carminator* (*Cryptalyra* Shaw: Neotropical) occur in far separated areas (Shaw 1990; Vilhelmsen et al. 2010). Further findings from Southeast Asia will help the understanding of the evolutionary history of the Megalyridae.

## Materials and methods

The morphological terms used in the descriptions follow Shaw and van Noort (2009) and Vilhelmsen et al. (2010). Photo images were produced using Leica Application Suite (Leica Microsystems) and Combine ZM software (Alan Hadley, www.hadleyweb.pwp.blueyonder.co.uk). Line drawings were made using a drawing tube attached to a binocular microscope. The depositories of the types are as referenced after the collection data.

## Taxonomy

### Genus Ettchellsia

Cameron, 1909

http://species-id.net/wiki/Ettchellsia

Ettchellsia : Cameron 1909: 208; Baltazar 1961: 219; He 1991: 475

#### Type species.

*Ettchellsia piliceps* Cameron, 1909 (monotypic).

#### Diagnosis.

**Figures 1–3.**
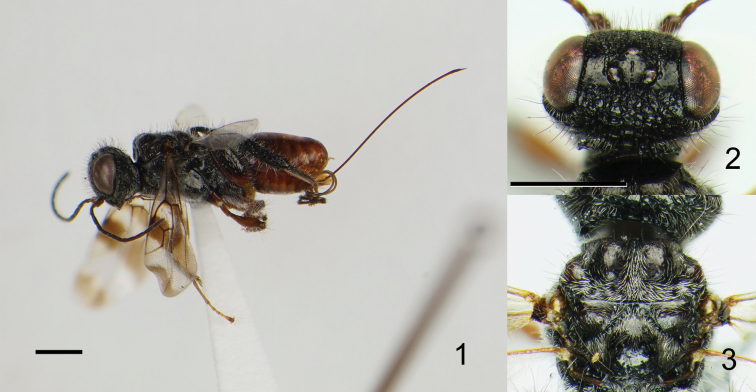
*Ettchellsia ignita*, sp. n. (holotype). **1** General habitus **2** Head in dorsal view **3** Mesosoma in dorsal view. Scale = 1.0 mm.

**Figures 4–6.**
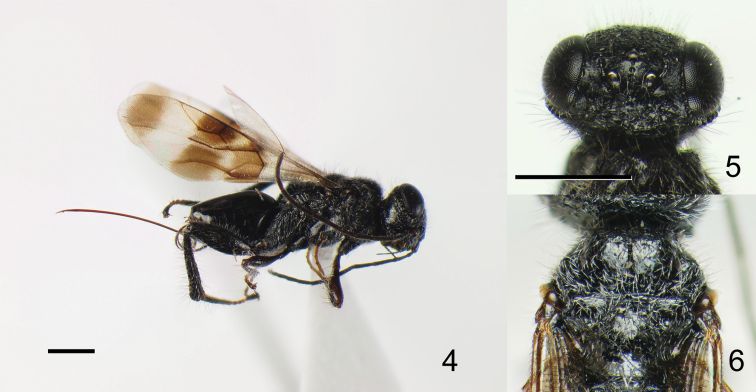
*Ettchellsia nigripes*, sp. n. (holotype). **4** General habitus **5** Head in dorsal view **6** Mesosoma in dorsal view. Scale = 1.0 mm.

**Figures 7–9.**
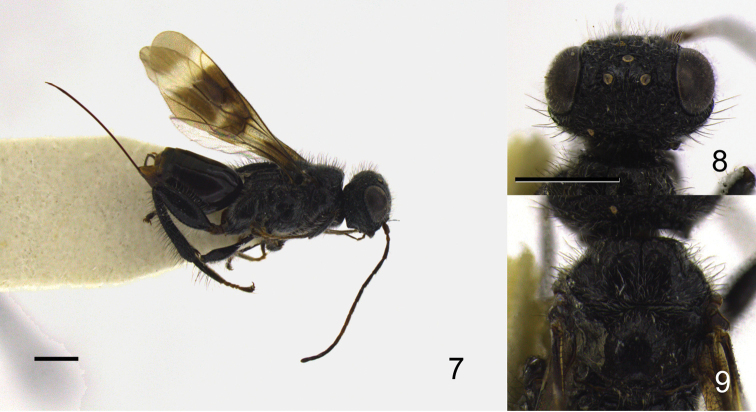
*Ettchellsia reidi*, sp. n. (holotype). **7** General habitus **8** Head in dorsal view **9** Mesosoma in dorsal view. Scale = 1.0 mm.

Eye margined posteriorly by post-orbital orbital carina; posterior border of mesopleuron smooth, without row of foveae; propodeum bearing unique pattern of longitudinal carinae (Figs 12–14); fuscous banding pattern present on fore wing (Figs 1, 4, 7) or wing entirely fuscous; fore wing with RS between RS+M and r-rs tubular (Fig. 4); apical part of RS tubular, arched toward stigma (Fig. 4); M+Cu and Cu1 spectral (Fig. 4); hind coxa bearing longitudinal carina; hind tibia rugose (Figs 1, 4, 7).

Detail generic character states were discussed by Shaw (1990) and Vilhelmsen et al. (2010).

#### Key to the females of the species of *Ettchellsia*

**Table:** 

1	Ocellar triangle smooth, without a longitudinal row of punctures; smooth crescent-shaped depression present on outer margin of each ocellus; wings mostly clear except with some infumation medially and apically, but lacking distinct dark bands	*Ettchellsia philippinensis* Baltazar
–	Ocellar triangle sculptured with at least one median longitudinal row of punctures (Fig. 2); crescent-shaped depressions absent near ocelli, or depressions irregularly foveate, not smooth; wings with three or four distinct dark bands	2
2	Vertex strongly flattened, appearing flat in lateral view (Fig. 11); mesonotum more weakly setose, setae not covering surface	*Ettchellsia piliceps* Cameron
–	Vertex well-developed and convex, appearing round in lateral view (Fig. 10); mesonotum medially and posteriorly with thick setae largely covering surface	3
3	Mesoscutum strongly humped (Fig. 3)	4
–	Mesoscutum not humped (Fig. 6)	5
4	Median region of propodeum anteriorly strongly narrowed (as in Fig. 12); hind tibia and basitarsi dorsally with long black erect setae, many of which are longer than the width of the hind tibia; metasoma reddish brown (Fig. 1)	*Ettchellsia ignita* Mita & Shaw, sp. n.
–	Median region of propodeum not anteriorly strongly narrowed; hind tibia and basitarsi dorsally with long erect white (not black) setae, most of which are shorter than the width of the hind tibia; metasoma black (as in Fig. 4)	*Ettchellsia sinica* He
5	Median region of propodeum medially narrowed (Fig. 14); vertex between posterior ocellus and eye rugose (Fig. 8)	*Ettchellsia reidi* Mita & Shaw, sp. n.
–	Median region of propodeum anteriorly weakly narrowed (Fig. 13); vertex between posterior ocellus and eye mostly smooth (Fig. 5)	*Ettchellsia nigripes* Mita & Shaw, sp. n.

### Ettchellsia ignita

Mita & Shaw, sp. n.

urn:lsid:zoobank.org:act:5CDAC4E0-568D-4F4A-A72B-63AA5A4051C2

http://species-id.net/wiki/Ettchellsia_ignita

Figs 1–3 10, 12

#### Type series.

Holotype ♀: “MALAYSIA: Negeri Selangor, Ulu Gombak (Univ. Malaya Field Studies Centre, 220m alt) Malaise trap, 7–11.iv.2007 T. Tsuru & M. Maruyama leg.”, “HOLOTYPE: *Ettchellsia ignita* Mita & Shaw, 2012, sp. n.”. Paratype: 1♂, Sandan, Borneo, Baker leg. The holotype is deposited in the Systematic Entomology, Hokkaido University Museum, Sapporo, Japan (SEHU).The paratype is deposited in the U.S. National Museum of Natural History, Washington D.C, USA (NMNH).

#### Description.

(Female)Head (Figs 1–2) 1.48 × wider than long, covered with long erect setae and short decumbent setae; frons reticulate; surface around ocellar triangle smooth with rows of punctures behind anterior ocellus and outside of posterior ocelli; vertex reticulate-rugose; eye margined posteriorly by foveate groove and single post-ocular orbital carina; gena sparsely punctate with irregular carinae under orbital carina; occipital carina forming a small depression; clypeus transversely rugose.

Mesosoma (Fig. 3) entirely covered with short decumbent white setae, but long erect setae also present on dorsal surface; pronotum dorsally forming acute corner; mesoscutum humped, sparsely scattered with small punctures; lateral carina present on anterior mesoscutal surface; axilla and scutellum sparsely scattered with small punctures; metanotum setose; propodeum (Fig. 12) with pair of median, submedian and lateral carinae; median carina wider than other carinae, dorsal surface flattened (Fig. 12); median propodeal region narrower anteriorly, with several transverse carinae, posterior margin dorsally produced; submedian region with three transverse carinae, medially narrowed; lateral region with four transverse carinae.

Fore wing (Fig. 1) bearing four transverse dark bands; vein M 1.9 × basal part of RS; erect setae on C 0.2 × those on Sc+R and A.

Metasoma smooth but anterior surface of 6th metasomal tergite and 7–8th tergites entirely strongly shagreened; ovipositor 2.00 × mesosoma length, apex with small teeth and single knob.

*Color*. Head black; mandible black; antenna brown-black except scape and pedicel brownish; long setae on vertex and gena black, other setae white. Mesosoma black except brown tegula; long erect setae black; fore- and middle legs brown; hind leg with coxa, distal half of femur and distal four tarsomeres brown-black, trochanter and basal part of femur brown, tibia and basitarsus black; long setae on dorsal surface of hind tibia and basitarsus black. Metasoma and ovipositor reddish brown; ovipositor sheath pale brown.

*Measurements*. Head 1.05 mm long, 1.55 mm wide; mesosoma 2.10 mm long; scutum 1.35 mm wide; propodeal disc 0.70 mm long, 1.10 mm wide; fore wing 4.75 mm long; metasoma 2.30 mm long, 1.25 mm wide; ovipositor 4.20 mm long; total body length excluding ovipositor 5.45 mm.

(Male) Different from female as follows: Body brownish, head dark brown with brown antenna, mesosoma brown, legs testaceous excluding brown hind tarsus, metasoma testaceous; median carinae on propodeum narrower, dorsal surface rounded (as in Fig. 13).

*Measurements*. Head 0.76 mm long, 1.24 mm wide; mesosoma 1.75 mm long; scutum 1.10 mm wide; propodeal disc 0.55 mm long, 0.90 mm wide; fore wing 3.55 mm long; metasoma 1.40 mm long, 0.95 mm wide; total body length 3.77 mm.

#### Distribution.

Peninsular Malaysia; Borneo.

#### Etymology.

This species is named for the reddish coloration of the metasoma.

#### Remarks.

This species is similar to *Ettchellsia sinica* with both having a strongly humped mesoscutum, however, it is distinguished from the latter by the strongly narrowed median region of propodeum (Fig. 12); long erect black setae on hind tibia and basitarsus, many of which are longer than the width of the hind tibia (setae are whitish and shorter in *Ettchellsia sinica*); reddish brown metasoma (Fig. 1) (metasoma is black in *Ettchellsia sinica*).

### Ettchellsia nigripes

Mita & Shaw sp. n.

urn:lsid:zoobank.org:act:1B9CC5DE-5AAC-483A-A952-B36A84C5DDFE\

http://species-id.net/wiki/Ettchellsia_nigripes

Figs 4–6 13

#### Holotype

♀: “N. Sulawesi: Prov. Gorontalo, Pegunungan Tilongkabila, Bogani Nani Warta Bone N.P., 31. Jan-16 Feb 2010 (alt. 1200m), K. Takasuka leg. (Malaise trap)”, “HOLOTYPE: *Ettchellsia nigripes* Mita & Shaw, 2012, sp. n.”. The holotype is deposited in the Laboratory of Entomology, Faculty of Agriculture, Tokyo University of Agriculture, Atsugi, Japan.

#### Description.

(Female)Head (Figs 4–5) 1.60 × wider than long, covered with long erect setae and short decumbent white setae; frons transversely rugose; surface around ocellar triangle smooth with rows of punctures behind anterior ocellus and outside of posterior ocelli; vertex reticulate-rugose; eye margined posteriorly by foveate groove and single post-ocular orbital carina; gena smooth with irregular carina under orbital carina; occipital carina not forming a depression; clypeus punctuate with small punctures.

Mesosoma (Fig. 6) entirely covered with short decumbent setae, but long erect setae also present on mesonotum; mesoscutum smooth except lateral carina present on anterior surface; dorsal mesoscutal surface not humped; axilla and scutellum smooth; metanotum setose; propodeum (Figs. 13) with pair of median, submedian and lateral carinae; median region narrowed anteriorly, with eight indistinct transverse carinae, posterior margin posteriorly produced; submedian propodeal region converging posteriorly, with two transverse carinae excluding posterior areola; lateral region with two (right side) or four (left side) transverse carinae.

Fore wing (Fig. 4) bearing four transverse dark bands but separation between second and third bands indistinct; vein M 2.2 × basal part of RS; erect setae on C 1.2 × longer than those on Sc+R, equal to those on vein A.

Metasoma smooth except anterior surface of 6–8th metasomal tergites shagreened; ovipositor 1.29 × mesosoma length, apex with small teeth and single knob.

*Color*. Body entirely black except tarsi and ovipositor sheath dark brown, ovipositor reddish brown; long setae on vertex, gena, mesonotum and dorsal surface of hind tibia and basitarsus black, other setae white.

*Measurements*. Head 0.80 mm long, 1.55 mm wide; mesosoma 2.15 mm long; scutum 1.35 mm wide; propodeal disc 0.75 mm long, 1.10 mm wide; fore wing 4.85 mm long; metasoma 1.90 mm long, 1.20 mm wide; ovipositor 2.75 mm long; total body length excluding ovipositor 4.85 mm.

(Male) Unknown.

#### Distribution.

Sulawesi Island.

#### Etymology.

This species is named for its black legs.

#### Remarks.

This species is similar to *Ettchellsia reidi* Mita & Shaw, sp. n. by the almost flat mesoscutum, however, it is distinguished from the latter by the anteriorly weakly narrowed median region of propodeum (Fig. 13) and the mostly smooth surface between posterior ocellus and eye (Fig. 5).

### Ettchellsia reidi

Mita & Shaw, sp. n.

urn:lsid:zoobank.org:act:403B45A6-E102-41E7-8A52-EBDC05C98BED

http://species-id.net/wiki/Ettchellsia_reidi

Figs 7–9 14

#### Type series.

Holotype ♀: “Sungai Sibau, nr entrance to Betung, Kerihun NP. ca. 4 km upstream, Kampung Putan. 21–27 Jun 1996, 70-90m. Chris Reid. IIS 967005”, “20 yr. old lading, closed forest, Pan traps (yellow), 1°03'13"N, 113°00'56"E”, “SEM”, “HOLOTYPE: *Ettchellsia reidi* Mita & Shaw, 2012”. Paratype: 1♀, same data as above, but Pan traps (yellow) 2:9, without label of “SEM”. The holotype is deposited in the Museum Zoologi Bogor (MZB), the national museum of Indonesia. The paratype is deposited in the Royal Ontario Museum (ROM) Toronto, Canada.

#### Description.

(Female) Head (Figs 7–8) 1.53–1.56 × wider than long, covered with long erect setae and short decumbent white setae; frons transversely rugose; surface around ocellar triangle rugose with rows of punctures behind anterior ocellus and outside of posterior ocelli, but lateral row indistinct among other sculptures; vertex reticulate rugose; eye margined posteriorly by foveate groove and single post-ocular orbital carina; gena smooth with irregular carinae under orbital carina; occipital carina dorso-laterally with a small depression; clypeus smooth.

Mesosoma (Fig. 9) entirely covered with short decumbent setae, but long erect setae also present on mesonotum; mesoscutum smooth; lateral carina present on anterior surface; dorsal surface not swollen; axilla and scutellum smooth; metanotum setose; propodeum (Fig. 14) with pair of median, submedian and lateral carinae; median propodeal region narrower centrally, with a few transverse carinae in holotype, carinae indistinct in a paratype; posterior margin dorsally producing; submedian region smooth excluding posterior areola, parallel-sided; lateral region with at most four transverse carinae but keels sometimes indistinct.

Fore wing (Fig. 7) bearing three transverse dark bands with clear spot around Rs+M; vein M 1.6–1.7 × basal part of RS; erect setae on C 2.0 × those on Sc+R, 1.2 × those on A.

Metasoma smooth except anterior surface of 6–8th metasomal tergites shagreened; ovipositor 1.34–1.45 × mesosoma length, apex with small teeth and single knob.

*Color*.Head black; mandible dark brown; antenna dark brown except scape, pedicel and 5–8th flagellomeres brown; long setae on vertex, gena black. Mesosoma black; long setae on mesonotum black; legs black except tarsi brownish; long setae on dorsal surface of hind tibia and basitarsus black. Metasoma brownish black; ovipositor and ovipositor sheath dark brown.

*Measurements*. Head 0.80–1.10 mm long, 1.25–1.65 mm wide; mesosoma 1.55–2.20 mm long; scutum 0.95–1.30 mm wide; propodeal disc 0.65–0.85 mm long, 0.85–1.10 mm wide; fore wing 3.50–4.90 mm long; metasoma 1.55–2.35 mm long, 0.85–1.25 mm wide; ovipositor 2.25–2.95 mm long; total body length excluding ovipositor 3.75–5.45 mm.

(Male) Unknown.

#### Distribution.

Kalimantan Barat, Borneo.

#### Etymology.

The species name is dedicated to the collector of the types, Chris Reid, a coleopterist working at the Australian Museum, Sydney.

#### Remarks.

The specimens were collected as part of the Insects of Indonesia Project, a collaboration of the ROM with the MZB. This species is similar to *Ettchellsia nigripes* Mita & Shaw, sp. n. About identification of the two species, see the remarks of *Ettchellsia nigripes*.

**Figures 10–14.**
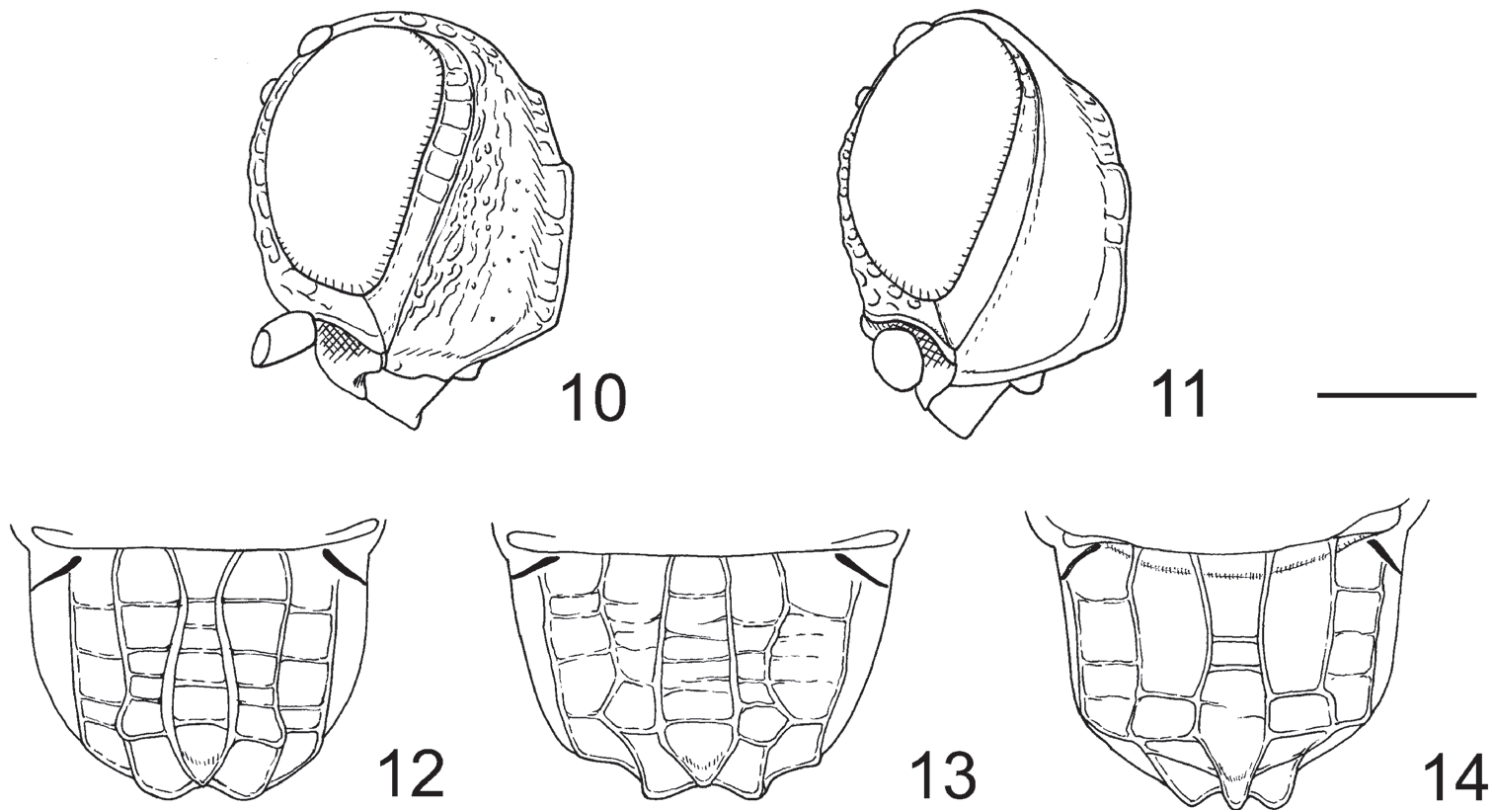
Heads and propodea of *Ettchellsia* spp. **10, 12** *Ettchellsia ignita*, sp. n. **11** *Ettchellsia piliceps* from Borneo **13** *Ettchellsia nigripes*, sp. n. **14** *Ettchellsia reidi*, sp. n. Scale = 0.5 mm.

## Supplementary Material

XML Treatment for Ettchellsia

XML Treatment for Ettchellsia ignita

XML Treatment for Ettchellsia nigripes

XML Treatment for Ettchellsia reidi
